# Appendectomy Mitigates Coxsackievirus B3−Induced Viral Myocarditis

**DOI:** 10.3390/v15101974

**Published:** 2023-09-22

**Authors:** Chengrui Niu, Wei Xu, Sidong Xiong

**Affiliations:** Jiangsu Provincial Key Laboratory of Infection and Immunity, Institute of Biology and Medical Sciences, Soochow University, Suzhou 215123, China; ncr1529404005@163.com

**Keywords:** appendectomy, CVB3, viral myocarditis, IL-10

## Abstract

Appendix has a distinct abundance of lymphatic cells and serves as a reservoir of microbiota which helps to replenish the large intestine with healthy flora. And it is the primary site of IgA induction, which shapes the composition of the intestinal microbiota. Recent population-based cohort studies report that appendectomy is associated with an increased risk of acute myocardial infarction and ischemic heart disease. Here, whether appendectomy has an effect on the occurrence and development of coxsackievirus B3 (CVB3)−induced viral myocarditis is studied. 10^3^ TCID_50_ CVB3 was inoculated i.p. into appendectomized and sham-operated mice. RNA levels of viral load and pro-inflammatory cytokines in the hearts and the intestine were detected by RT−PCR. Compared to sham-operated mice, appendectomized mice exhibited attenuated cardiac inflammation and improved cardiac function, which is associated with a systemic reduced viral load. Appendectomized mice also displayed a reduction in cardiac neutrophil and macrophage infiltration and pro-inflammatory cytokine production. Mechanistically, we found that CVB3 induced an early and potent IL-10 production in the cecal patch at 2 days post infection. Appendectomy significantly decreased intestinal IL-10 and IL-10^+^ CD4^+^ Treg frequency which led to a marked increase in intestinal (primary entry site for CVB3) anti-viral IFN-γ^+^ CD4^+^ T and IFN-γ^+^ CD8^+^ T response and viral restriction, eventually resulting in improved myocarditis. Our results suggest that appendix modulates cardiac infection and inflammation through regulating intestinal IL-10^+^ Treg response.

## 1. Introduction

The appendix has been considered a redundant organ in the human body for decades. Now, it is considered an important part of the gut-associated lymphoid tissue (GALT). It contains dense lymphoid tissue resembling Peyer’s patches and is the primary site for immunoglobulin A production which is crucial to regulate the density and quality of the intestinal flora [1]. There are many lymphoid follicles in the submucosa of the appendix, which are rich in B and T lymphocytes and macrophages [2]. The appendix is also a unique reservoir of highly diverse microbiome [3] and can serve as a “safe house” for normal microbiota [4]. Therefore, the appendix might play an important role in intestinal immunity by maintaining the microbiota as a reservoir, and by inducing or modulating intestinal immune response.

With the deepening understanding of the function of the appendix, a large number of studies have shown that it plays an important role in intestinal immunity and maintaining the dynamic equilibrium of intestinal microbiota [5]. The appendix has a certain impact on the occurrence, development and prognosis of intestinal and extraintestinal diseases [6]; recent studies have found that the appendix could contribute to the pathogenesis of inflammatory bowel diseases (IBD), in particular ulcerative colitis (UC) [4,7]. The appendix may play a role in the pathogenesis of Parkinson’s disease (PD). Some studies reported that appendectomy due to appendicitis was associated with a reduction in risk for developing PD [8]. Appendectomy was related to the increased risk of many autoimmune diseases. Data statistics found that the incidence of systemic lupus erythematosus (SLE) was higher in patients receiving appendectomy [9]. Appendectomy was also related to the cardiovascular diseases, as a highly publicized recent study showed. It was found that the removal of the appendix before the age of 20 was associated with a higher risk of acute myocardial infarction [10]’ another study found a 1.54 times greater risk of ischemic heart disease within three years after appendectomy [11]. The above findings indicate that appendectomy may have a certain impact on the occurrence and development of many diseases. Further investigations are required to explore its role in diseases and clarify its potential value.

Viral myocarditis (VMC) is a common clinical cardiovascular disease [12]; it is the main cause of sudden death in children and adolescents [13]. VMC is characterized by diffuse inflammatory infiltration of myocardial tissue after viral infection [14], and can further develop into life-threatened dilated cardiomyopathy [15]. Infection of coxsackievirus B3 (CVB3), one of enteroviruses, is an important causing factor for VMC. CVB3 is naturally transmitted through the oral–fecal route; it infects the GI epithelium with limited replication and then disseminates into the heart and pancreas [16]. Experimentally, however, the oral route of CVB3 infection results in low morbidity and mortality rates with only mild disease [17]. Thus, intraperitoneal route is commonly employed in pathogenetic studies, and the resulting viremia induces mild myocarditis and severe pancreatitis in mice [16]. Since the intestine is the primary target organ for CVB3 infection, CVB3−induced intestinal immunity may have an impact on the systemic inflammatory response, thus affecting the occurrence of VMC [16].

Despite the fact that mice lack a separate appendix, they have a cecal patch rough equivalent to the human appendix [18]. Compared to multiple small lymphoid clusters in the human appendix, the cecal patch contains a single large lymphoid cluster [19] and serves as a secondary lymphoid organ [20]. Whether appendectomy may affect the risk of CVB3−induced myocarditis remains unclarified.

In this study, we aimed to clarify the role of the appendix in the occurrence and development of viral myocarditis. We found that appendectomy reduced the viral load and the incidence as well as severity of VMC, which may provide a more comprehensive understanding of the regulatory function of appendix.

## 2. Materials and Methods

### 2.1. Virus and Animals

CVB3 virus (Nancy strain) was maintained in our lab and passaged in HeLa cells. HeLa cells were cultured in Dulbecco’s modified Eagle’s medium (DMEM) supplemented with 10% FBS at 37 °C in 5% CO_2_ [21].

Male BALB/c mice aged 4–5 weeks were purchased from Gempharmatech Co., Ltd. (Nanjing, China), and housed in the SPF condition. All animal experiments were performed with the approvement of the Laboratory Animal Center and the Ethics Committee of Soochow University.

### 2.2. Surgical Procedures

Two different surgical procedures were performed under general anesthesia (intraperitoneal injection of Avertin at 0.2 mL/10 g): appendectomy and sham operation [1,22,23]. The left abdomen of the mice was shaved after anesthetization, covered with a sterile cloth and disinfected with a 75% ethanol; a longitudinal incision of 0.5–1 cm was performed and the peritoneum was further cut. The cecum was externalized from the peritoneal cavity; the cecal patch was identified as the white lymphoid patch at the end of the cecum. The base of the cecal patch was ligated with suture and resected to ensure complete removal of lymphoid tissue. The cecum was kept moist with preheated sterile physiological saline during operation. Then, the peritoneum and skin were closed. Sham-operated control mice underwent the same procedure without the removal of the cecal patch. After 1 h of surgical procedure, both groups of mice were observed on the subject of recovery, and weight was monitored daily during one week.

### 2.3. CVB3−Induced Viral Myocarditis in Mice

Two groups of mice that underwent surgery (appendectomy and sham operation) were intraperitoneally injected with 10^3^ TCID_50_ of CVB3 one week later. The development of acute myocarditis was evaluated according to cardiac viral load, myocardial inflammation, cardiac function, and cardiac troponin I (cTnI) seven days post CVB3 infection as described previously [24].

### 2.4. Echocardiography

The cardiac function of the infected mice was evaluated using the Visual Sonics Vevo 2100, a high-resolution ultrasound imaging system. Two-dimensional guided M-mode echoes were obtained from short- and long-axis views at the level of the largest left ventricular (LV). The LV ejection fraction (LVEF) and fractional shortening (LVFS) were calculated.

### 2.5. RT−PCR

Total RNA was extracted with the TRIzol reagent and then reversely transcribed to cDNA, which was subjected to RT−PCR with the SYBR green system (Takara) using the specific primers shown in Table 1.

### 2.6. Histopathological Analysis

Hearts were collected at seven days post CVB3 infection after appendectomy and sham operation, fixed in a 4% paraformaldehyde and embedded in paraffin. Paraffin-embedded sections (5 μm) were deparaffinized and stained with a hematoxylin and eosin (H&E) reagent. The inflammatory pathological scoring was based on the extent of inflammatory cell infiltration and myocardial necrosis area as previously described: a score of 0 showed no infiltration of inflammatory cells or myocardial necrosis; 1 point manifested as inflammatory cell infiltration range and myocardial necrosis area < 25%; 2 points showed infiltration of inflammatory cells and myocardial necrosis area ranging from 25% to 30%; a score of 3 indicated an infiltration range of inflammatory cells and a myocardial necrosis area of 50% to 75%; a score of 4 indicated the infiltration range of inflammatory cells and myocardial necrosis area > 75% [25]. Sections were scored in a blinded manner by two investigators separately.

### 2.7. Isolation of Cells from Cecal Patch and Flow Cytometry

The cecal patch was isolated and washed with PBS, then gently ground using the plunger of a 5 mL syringe. The cell suspension was passed through a 70 μm cell strainer and collected for flow cytometry after centrifugation. The cells were incubated with Pacific Blue anti-CD45 (BioLegend, San Diego, CA, USA), PE anti-CD4 (BD Biosciences, San Jose, CA, USA), PerCP-Cy5.5 anti-CD8 (BioLegend), APC anti-F4/80 (BioLegend), PE anti-B220 (BD Biosciences) mAbs for 30 min at 4 °C. The samples were washed twice with PBS, then acquired using BD FACS Canto II and analyzed with FlowJo v10.

### 2.8. Isolation of Immune Cells from Intestinal Lamina Propria and Heart

The intestines were incubated in HBSS containing 5  mM EDTA at 37 °C with shaking for 20 min [1,26], and then cut into small pieces, digested with RPMI 1640 containing 10% FBS, collagenase IV (100  mg/mL, Yeasen, Shanghai, China), DNase I (50 mg/mL, Yeasen) at 37 °C for 1 h in a thermostatic oscillator. Fresh heart tissues were washed with cold PBS and cut into small pieces, then digested with RPMI 1640 containing 10% FBS, collagenase II (50 U/μL, Gibco, New York, NY, USA), and hyaluronidase (30 mg/mL, Sigma, Livonia, MI, USA) at 37 °C for 1 h.

The digested tissues were filtered through a 70 μm cell strainer. The cells were resuspended in a discontinuous 40/80% Percoll gradient at 500 g for 30 min. The immune cells were collected at the interface of the Percoll gradient.

### 2.9. Intracellular Cytokine Staining

The immune cells were incubated with phorbol myristate acetate (50 ng/mL, BioGems), ionomycin (1 μg/mL, BioGems, Westlake Village, CA, USA) and Brefeldin A (5 μg/mL, BioGems) at 37 °C for 5 h. After fixation and permeabilization using a Cytofix/Cytoperm kit (BD Biosciences), the cells were incubated with APC anti-IL-10 (BioLegend) mAbs at 4 °C for 45 min. The samples were washed twice with PBS, then acquired using BD FACS Canto II and analyzed with FlowJo v10.

### 2.10. Statistical Analysis

Statistical analyses were performed using GraphPad Prism 8.0 and presented as mean and standard error (mean ± SEM). Differences between control and experimental groups were evaluated using student’s *t*-test. One-way ANOVA was used to perform the intra-group comparisons among the experimental groups. *p* < 0.05 was considered statistically significant.

## 3. Results

### 3.1. Appendectomy Alleviated the Incidence and Severity of CVB3−Induced Myocarditis

**Figure 1:**
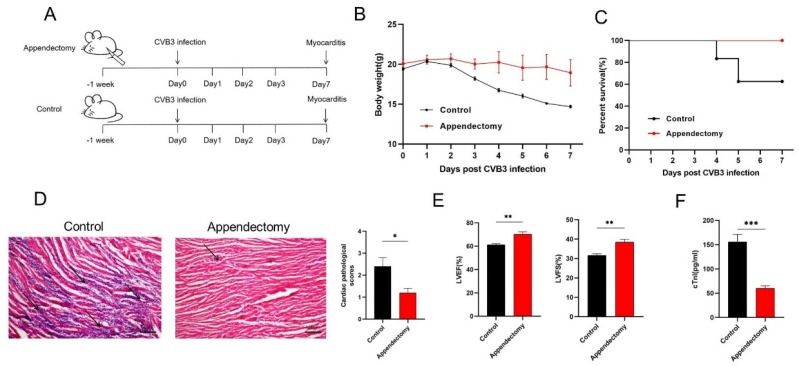
Appendectomy alleviated the incidence and severity of CVB3−induced myocarditis. (**A**) Experimental schema of appendectomy and control group (sham operation) infected with CVB3 used to induce myocarditis in mice. (**B**) The weight change and (**C**) survival rate of mice was followed during 7 days of CVB3 infection. (**D**) H&E staining and pathological scoring of heart sections showing inflammatory infiltration after CVB3 infection for 7 days. Scale bar: 100 μm. The arrows point to the site of inflammatory infiltration. (**E**) Cardiac function quantitative statistics of LVEF and LVFS. (**F**) Serum cTnI level. Values are presented as means ± SEM. * *p* < 0.05, ** *p* < 0.01, *** *p* < 0.001.

To investigate the impact of appendectomy on CVB3−induced VMC, mice were subjected to appendectomy and sham operation (control), and recovered for one week (Figure 1A). It was found that the surgical procedure did not affect the normal life activities and weight gain of mice. Then, the mice were intraperitoneally injected with a 1000 TCID_50_ dose of CVB3, and their survival and weight loss were monitored during 7 days of infection. Compared to a 15% weight loss seen in the control mice, appendectomized mice had almost no change in weight (Figure 1B). In total, 40% of the control mice died on 7 days post infection, while 100% appendectomized mice survived through 7 days (Figure 1C). HE staining of the cardiac section showed that the control mice hearts contained several focal necrosis and intensive inflammatory immune cell infiltration (pathological score 2.4), while appendectomized mice had largely reduced focal necrosis and immune infiltration in their hearts (pathological score 1.0, *p* < 0.05, Figure 1D). Consistent with this result, when cardiac function was evaluated by left ventricular ejection fraction (LVEF) and left ventricular fractional shortening (LVFS), we found that appendectomized mice had significantly increased LVEF and LVFS values compared with those of the control mice (Figure 1E). Appendectomized mice had significantly diminished cTnI serum levels (Figure 1F), indicating reduced cardiac injury. Taken together, our data indicate that appendectomy surgery alleviates CVB3−induced acute myocarditis and injury.

### 3.2. Appendectomized Mice Had Decreased Viral Replication in the Hearts and Small Intestine

**Figure 2:**
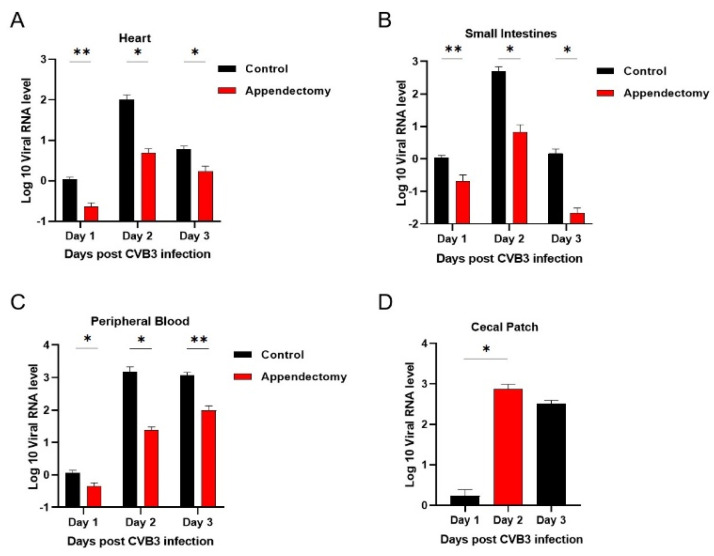
Appendectomized mice had decreased viral replication in the hearts and small intestine of mice. Viral RNA levels in the heart (**A**), intestine (**B**), blood (**C**) and cecal patch (**D**) were measured by RT−PCR. Values are presented as means ± SEM. * *p* < 0.05, ** *p* < 0.01.

To clarify whether the reduced cardiac injury and inflammation seen in the appendectomized mice is due to reduced viral replication, viral RNA level in hearts of mice on Day 1, 2 and 3 p.i. was detected by RT−PCR. The relative viral RNA levels in the hearts of mice were significantly decreased on Day 1, 2 and 3 p.i. after appendectomy (Figure 2A). CVB3, an enterovirus, infects the intestine epithelium before dissemination into the pancreas and heart [16]. The gut intestine is the primary entry site for CVB3 natural infection and i.p. infection [17,27]. Therefore, we measured the viral load in the intestine and found that the intestinal CVB3 RNA level was significantly diminished in the appendectomized mice compared to the control mice (Figure 2B). Intraperitoneal injection of CVB3 facilitates peripheral dissemination of the virus [28]. Viral RNA level in the blood of appendectomized mice was measured and also had a reduced level (Figure 2C). Therefore, appendectomized mice had significantly decreased viral load in the intestine, peripheral blood and heart at the early stage of infection, leading to reduced cardiac infection, injury and inflammation.

### 3.3. Appendectomized Mice Had Reduced Cardiac Infiltration of Neutrophils and Macrophages and Pro-Inflammatory Cytokine Production

**Figure 3:**
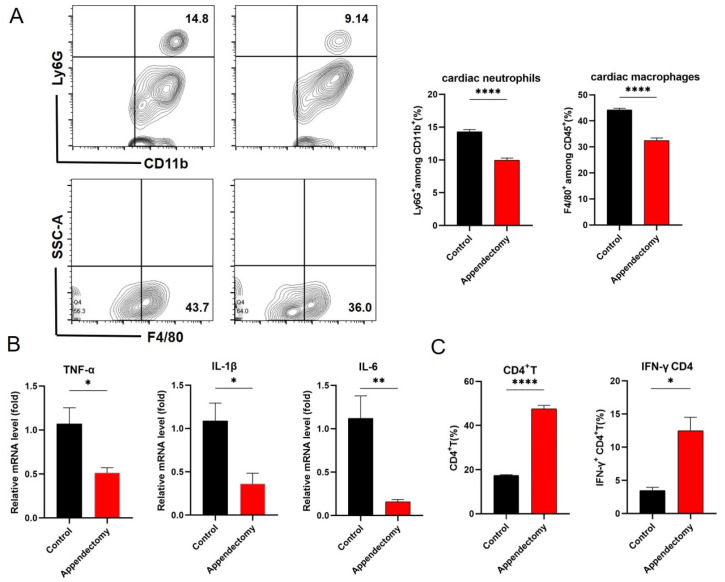
Appendectomized mice had reduced cardiac infiltration of neutrophils and macrophages and pro-inflammatory cytokine production. (**A**) Representative FACS plots and proportions of Ly6G^+^ and F4/80^+^ cells in the heart were measured by flow cytometry. (**B**) RNA levels of inflammatory cytokines in the heart were measured by RT−PCR. (**C**) Proportions of CD4^+^ T cells and IFN-γ^+^ CD4^+^ T cells in the heart were measured by flow cytometry. Values are presented as means ± SEM. * *p* < 0.05, ** *p* < 0.01, **** *p* < 0.0001.

To elucidate the mechanism of appendectomy-resulting reduction in CVB3-myocarditis and viral replication, it is necessary to analyze the immune cell composition and activation condition in the infected hearts and the intestine. After CVB3 infection, various cardiac-resident cells may contribute to acute inflammation by secreting cytokines such as TNF-α, IL-1β and IL-6 [29]. As the infection progresses, various innate immune cells (macrophages, NK cells, neutrophils) and adaptive immune cells (T cells and B cells) infiltrate and contribute to tissue damage by exerting cytotoxicity and secreting inflammatory cytokines [16]. Heart infiltrated mononuclear cells were isolated and analyzed by flow cytometry. It was found that at day 7 post CVB3 infection, significantly reduced proportions and numbers of CD11b^+^ Ly6G^+^ neutrophils (14.8% to 9.9% within CD45^+^ immune cells) and F4/80^+^ macrophages (44.5% to 31.8% within CD45^+^ immune cells) were evidenced in the hearts of appendectomized mice compared to those in the control mice (Figure 3A). Heart-infiltrated neutrophils and macrophages are the main producers of early pro-inflammatory cytokines; therefore, RNA levels of pro-inflammatory cytokines in the heart were detected. It was found that appendectomized hearts had significantly decreased levels of TNF-α, IL-1β and IL-6 than control mice hearts (Figure 3B). To see whether reduced viral replication was due to increased anti-viral response, cardiac CD4^+^ T cell and IFN-γ^+^ CD4^+^ T responses were detected. It was found that the proportions of CD4^+^ T cells were significantly increased, together with a significantly increased proportions of IFN-γ^+^ CD4^+^ T cells in the hearts of appendectomized mice than those in the control mice (Figure 3C). Taken together, appendectomized mice reduced cardiac immune cell infiltration and cytokine secretion in the heart.

### 3.4. Appendectomy Significantly Increased IFN-γ^+^ CD4^+^ T and IFN-γ^+^ CD8^+^ T Response in the Intestine after CVB3 Infection

**Figure 4:**
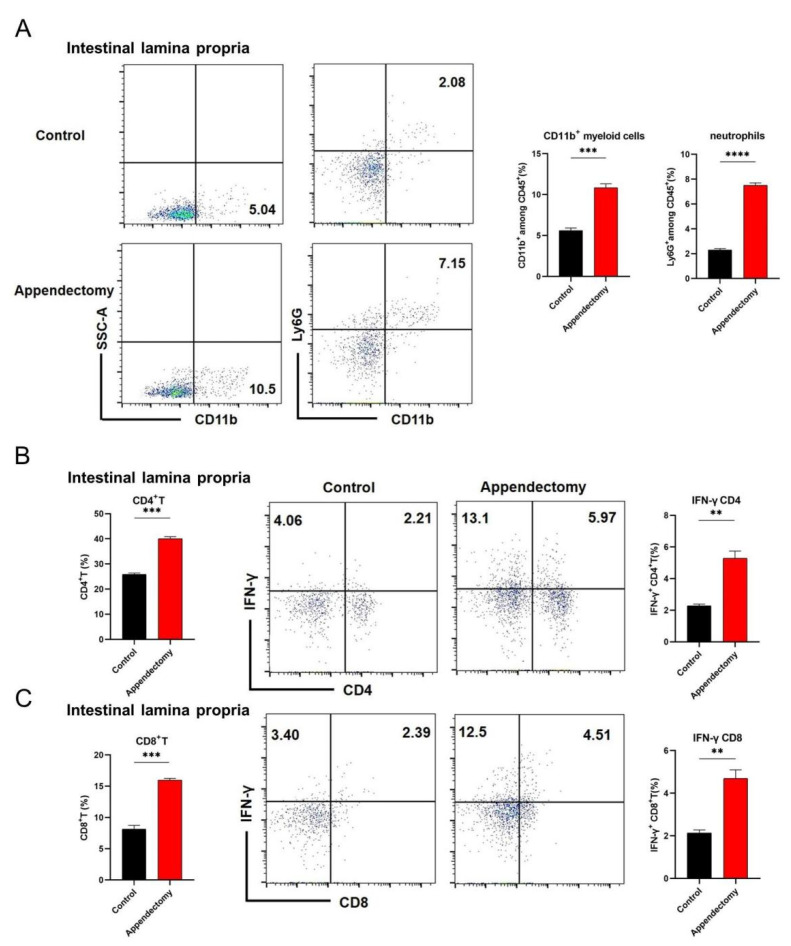
Appendectomy significantly increased IFN-γ^+^ CD4^+^ T and IFN-γ^+^ CD8^+^ T response in the intestine after CVB3 infection. (**A**) Proportions of CD11b^+^ cells and neutrophils in the intestinal lamina propria at 3 days p.i. were measured by flow cytometry. (**B**) Proportions of CD4^+^ T cells and IFN-γ^+^ CD4^+^ T cells in the intestinal lamina propria at 7 days p.i. were measured by flow cytometry. (**C**) Proportions of CD8^+^ T cells and IFN-γ^+^ CD8^+^ T cells in the intestinal lamina propria at 7 days p.i. were measured by flow cytometry. Values are presented as means ± SEM. ** *p* < 0.01, *** *p* < 0.001, **** *p* < 0.0001.

To elucidate the mechanism of appendectomy−induced cardiac immune infiltration reduction after CVB3 infection, it is necessary to reveal the immune cell alteration in the intestine, since appendectomy first influences immune cell composition and activation in the intestine. The intestine provides places between enteric viruses and the host [30]; it contains a large number of immune cells, especially T cells. Intestinal CD4 T cells and CD8 T cells play important functions in viral clearance discovered in murine studies [31]. By flow cytometry, we observed that the percentages of CD11b^+^ cells and neutrophils (Figure 4A) in the intestinal lamina propria were significantly increased in the appendectomized mice compared to the control mice at 3 days p.i. At 7 days p.i., we found that the percentages of CD4^+^ T cells and IFN-γ^+^ CD4^+^ T cell frequencies in the intestinal lamina propria were significantly increased in the appendectomized mice compared to the control mice (Figure 4B). Meanwhile, CD8^+^ T cells and IFN-γ^+^ CD8^+^ T cell frequencies in the intestinal lamina propria of appendectomized mice were also markedly increased compared to those in the control mice (Figure 4C). All the above data indicate that after appendectomy, the proportions and anti-viral function of intestine IFN-γ^+^ CD4^+^ T and IFN-γ^+^ CD8^+^ T cells were markedly increased, which may help these cells to clean the intestinal viruses.

### 3.5. Appendectomy Significantly Decreased Intestinal IL-10 Level and IL-10^+^ CD4^+^ Th Frequency after CVB3 Infection

**Figure 5:**
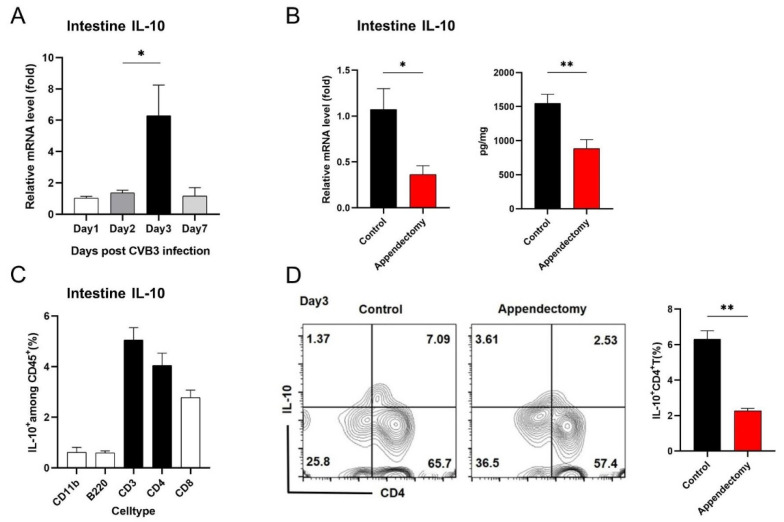
Appendectomy significantly decreased intestinal IL-10 level and IL-10^+^ CD4^+^ Th frequency after CVB3 infection. (**A**) RNA levels of IL-10 in the intestine after CVB3 infection were measured by RT−PCR. (**B**) RNA levels and protein levels of IL-10 in the intestine between two groups of mice were measured by RT−PCR and ELISA. (**C**) IL-10^+^ cells among CD45^+^ cells in the intestinal lamina propria after CVB3 infection were measured by flow cytometry. (**D**) FACS plots and proportions of IL-10^+^ CD4^+^ T cells in the intestinal lamina propria after CVB3 infection were measured by flow cytometry. Values are presented as means ± SEM. * *p* < 0.05, ** *p* < 0.01.

Why did appendectomy increase intestinal IFN-γ^+^ CD4^+^ T cell frequency? We suppose there might be an alteration in regulatory T cells. IL-10, an immune-brake cytokine, plays a key role in regulating the intestinal inflammatory response [32]; it has multiple immunosuppressive functions of immune responses to intestinal antigens [33]. First, we demonstrated that intestinal IL-10 peaked on Day 3 p.i. (Figure 5A). Then, by detecting IL-10 expression in the intestine, we found that both RNA and protein levels of IL-10 in intestine were significantly reduced in the appendectomized mice compared to in the control mice on Day 3 p.i. (Figure 5B). Flow cytometry analysis indicated that CD4^+^ T cells were the main producers of intestinal IL-10 (Figure 5C). And appendectomized mice had a markedly reduced IL-10-secreting CD4^+^ Treg frequency in the intestine compared to that of control mice (Figure 5D). These results indicate that the appendectomy-resulting intestinal IL-10 and IL-10^+^ Treg reduction contributes to increased intestinal IFN-γ^+^ CD4^+^ Th1 response.

### 3.6. Cecal Patch CD4^+^ T Cells Are Main IL-10 Producers after CVB3 Infection and Appendectomy May Lead to Intestinal IL-10 Reduction

**Figure 6:**
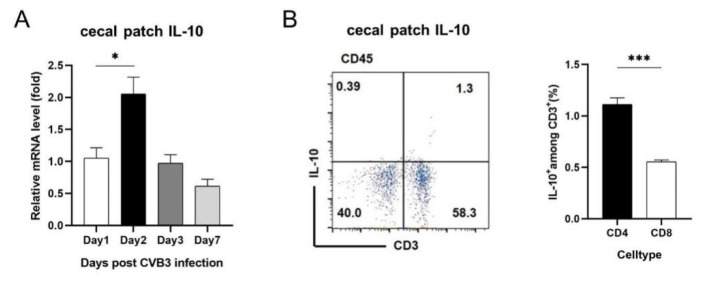
Cecal patch CD4^+^ T cells are main IL-10 producers after CVB3 infection. (**A**) RNA levels of IL-10 in the cecal patch after CVB3 infection were measured by RT−PCR. (**B**) FACS plots of IL-10^+^ CD3^+^ T cells and proportions of IL-10^+^ CD4^+^ T and CD8^+^ T cells among CD3^+^ T cells in the cecal patch after CVB3 infection were measured by flow cytometry. Values are presented as means ± SEM. * *p* < 0.05, *** *p* < 0.001.

Where does intestinal IL-10 come from? And why did appendectomy lead to intestinal IL-10 reduction? First, we detected the time course of IL-10 expression in the cecal patch. We found that IL-10 expression in cecal patch was significantly increased and peaked on Day 2 p.i. (Figure 6A), which seems preceding the intestinal IL-10 production. Then, we examined IL-10 producers in the cecal patch after CVB3 infection. After CVB3 infection, CD3^+^ Th cells were major IL-10 producers in the cecal patch, and CD4^+^ Th cells mostly contributed to cecal patch IL-10 production (Figure 6B). These data may explain why after appendectomy, intestinal IL-10 and IL-10^+^ CD4^+^ T cell frequencies were significantly decreased compared to those of control mice. Thus, appendectomy of mice led to increased anti-viral IFN-γ^+^ Th1 immune response in the intestine, decreased viral replication and reduced cardiac injury and myocarditis via down-regulating intestinal regulatory IL-10^+^ Treg cells.

Taken together, our data suggest that the early induced IL-10 and IL-10^+^ CD4^+^ Tregs in the cecal patch after CVB3 infection intimately regulate intestinal IL-10 and IL-10^+^ CD4^+^ Tregs situation, thus exerting an important pro-viral and pro-inflammatory effect in the development of viral myocarditis.

## 4. Discussion

Studies have shown that appendectomy has an impact on the occurrence and development of multiple diseases. The immune response caused by changes in immune function after appendectomy, such as a decrease in immunoglobulins, may affect the risk of cardiovascular disease [10]. Impaired immune system function may affect the risk of immune-system-related diseases such as ulcerative colitis, rheumatoid arthritis and ischemic heart disease [11], further deepening the connection between inflammation and cardiovascular disease. Recent studies have found that the human appendix plays a crucial role in facing with antigens from food sources and pathogens, and functions as the immune cell priming site; appendectomy has been reported to be associated with the occurrence and development of many diseases. Our studies first link appendix with the development of CVB3−induced viral myocarditis. And we found that the incidence and severity of viral myocarditis were markedly reduced by appendectomy. This result seems a little bit different from others that show that appendix removal was associated with a higher risk of acute myocardial infarction [10] and with an increased risk of ischemic heart disease within three years after appendectomy [11]. The above two relationships between appendix and cardiovascular diseases were based on human data. Human appendix function may not always be parallel with mice cecal patch function. And the above clinical cardiovascular diseases most probably have no relationship with viral infection; this study, in turn, focuses on the role of mice cecal patch in regulating virus−induced acute myocarditis. From our data, mice cecal patch intensively modulates intestinal IL-10^+^ CD4^+^ Treg induction and IL-10 production physiologically and pathologically, thus profoundly impacting viral infection and replication in the intestine (primary entry site for CVB3), then further modulating the onset of viral myocarditis. Although further exploration is needed to confirm all the above findings due to research limitations, it is necessary to reassess the necessity of surgical appendectomy with respect to treatment of cardiovascular diseases.

Viral myocarditis poses a certain threat to global health, especially in children and adolescents, and its pathogenesis is not yet fully understood [34]. CVB3 is an enteric virus that initiates infection in the gastrointestinal tract before disseminating to peripheral tissues to cause disease, but intestinal factors that influence viral replication are understudied [16]. The intestine, the initial target organ for CVB3 infection, has been paid much attention to due to its regulation in the pathogenesis of myocarditis. In the present study, we explore the role of the appendix, one important intestinal region, in the occurrence and development of viral myocarditis for the first time. Our data indicate that the incidence and severity of myocarditis are markedly alleviated in the appendectomized mice compared to those of control mice. RNA levels of cardiac pro-inflammatory cytokines are significantly reduced (Figure 3B) at 7 days post infection. Proportions and numbers of cardiac infiltrated neutrophils and macrophages are also significantly decreased (Figure 3A) in the appendectomized mice. Previous studies have shown that the direct damage of viruses to myocardial cells is one of the causes of VMC, and the viral load of the heart can be a driving factor of myocarditis [35]. In this regard, we detect the viral RNA level in the intestine and heart, and find that viral levels are significantly reduced in all tissues (heart, intestine, blood, Figure 2) of the appendectomized mice compared to those of the control mice during the acute phase of infection. Therefore, appendectomy mitigates CVB3−induced myocarditis through reducing viral infection intestinally and systemically.

What contributes to the reduced viral replication after appendectomy of mice? We then identify a substantial increase in the intestinal IFN-γ^+^ CD4^+^ and IFN-γ^+^ CD8^+^ T cell responses (Figure 4B, C) in the appendectomized mice, which could explain the reduction in intestinal viral load. IFN-γ is mainly secreted by CD4^+^ Th1 cells and cytotoxic CD8^+^ T cells, and is a crucial effector in anti-viral response [36]. As a kind of pleiotropic cytokine, IFN-γ has a non-specific antiviral effect, enhancing the killing effect of immune cells against pathogens, and collaborating with other immune cells to kill virus infected cells. Its immune regulatory activity plays a critical role in determining the long-term antiviral status of the body compared to other interferons [37].

Previous studies have found that IL-10 acts as an inducible immunoregulatory factor that can be generated in the condition of inflammatory demand [38]. Studies have suggested that the increase in IL-10 may be caused by the downregulation feedback of acute inflammation [39]. Animal studies implicate that IL-10, specifically CD4^+^ T-cell-derived IL-10, is a necessary mediator of intestinal immune homeostasis [40]. It also plays an immunostimulatory role in enhancing antibody production and cytotoxicity [41]. We find that CVB3 infection induced a robust up-regulation of IL-10 in the intestine (Figure 5A) and cecal patch (Figure 6A) at 3 days post infection. And CD4^+^ T cells proved to be the main producers of the above IL-10 (Figure 5C and Figure 6B). Of note, after the removal of the cecal patch, intestinal IL-10 and intestinal IL-10^+^ Treg were both significantly decreased (Figure 5B,D). This suggests that intestinal CD4^+^ Treg and IL-10 production is intensively regulated by cecal patch CD4^+^ Treg. Owing to their potent anti-inflammatory activity, decreased intestinal IL-10 and intestinal IL-10^+^ Treg led to increased intestinal IFN-γ^+^ T cell response, which contributed to viral restriction in the intestine first, then blood and heart, eventually resulting in mitigated myocarditis as well as reduced cardiac cytokine production (TNF-α, IL-1β, IL-6, Figure 3B). Cecal patch may therefore represent an important immunoregulatory organ and have deep effect on the occurrence and development of enterovirus-related myocarditis.

Innate immunity is the first line of defense against enterovirus infection. However, the role of neutrophils in viral infections remains largely understudied, particularly in the myocardium, where they are recruited in huge numbers at a very early phase of viral infection (Days 1–3 post infection). Neutrophils display diverse phenotypes and functions that can assist in viral clearance or augment and amplify the immunopathology of viruses. Neutrophils could produce proinflammatory factors such as IL-17A, IFN-γ, macrophage inflammatory protein (MIP-1α/β), which further activate function of macrophages and NK cells. Activated macrophages and NK then influence neutrophil function through secretion of GM-CSF, TNF-α and IL-1β [42]. In this study, we demonstrate that the cecal patch is a very important organ to regulate intestinal IL-10^+^ Treg induction and IL-10 production during an early phase of CVB3 infection. The appendectomized mice had decreased IL-10^+^ Treg and IL-10 level in the intestine, resulting in an increased intestinal IFN-γ^+^ T cell response, together with an increased percentage of CD11b^+^ Ly6G^+^ neutrophils (Figure 4A) in the intestinal lamina propria at 3 days p.i. Increased intestinal IFN-γ^+^ T cell response led to viral restriction both in the intestine and in the heart. Reduced viral load in the heart is usually associated with reduced neutrophil cardiac infiltration. That is why we find substantially reduced proportions and numbers of CD11b^+^ Ly6G^+^ neutrophils (14.8% to 9.9%) at Day 7 post infection in the hearts of appendectomized mice compared to those in the control mice (Figure 3A). The precise role of organ-infiltrated neutrophils in CVB3 infection is yet to be determined in terms of their phenotype, phagocytosis capacity and pro-inflammatory roles.

Collectively, our results indicate that the appendix is an important immune-modulatory organ or region to regulate the induction and maintenance of intestinal IL-10^+^ CD4^+^ Treg response and IL-10 production. In response to enterovirus infection, the appendix has an important impact on the control of viral replication locally and systemically, thus determining the onset and severity of viral myocarditis. Removal of the appendix may have a profound influence on the intestinal anti-viral immunity, and extra-intestinal anti-viral immunity. Our findings provide a comprehensive understanding of the association between the appendix and the heart, and indicate that appendectomy, which removes intestinal mucosa-associated lymphoid tissue, may alter the subsequent cardiovascular risk.

## Figures and Tables

**Table 1:** Primers used in RT−PCR assay.

Genes	Sequence (5′-3′)
*Actin*	F: CACTGTCGAGTCGCGTCCA R: TGACCCATTCCCACCATCAC
*CVB3*	F: AACGCCAAAACAACGGATGG R: GATCTGGGTCTGGGGGTAGT
*TNF-α*	F: CCCTCACACTCAGATCATCTTCT R: GCTACGACGTGGGCTACAG
*IL-1β*	F: GCAACTGTTCCTGAACTCAACT R: TCTTTTGGGGTCCGTCAACT
*IL-6* *Il-10*	F: TAGTCCTTCCTACCCCAATTTCC R: TTGGTCCTTAGCCACTCCTTC F: GCTCTTACTGACTGGCATGAG R: CGCAGCTCTAGGAGCATGTG

## Data Availability

The data presented in this study are available on request from the corresponding author.
